# Isolation of anti-toxin single domain antibodies from a semi-synthetic spiny dogfish shark display library

**DOI:** 10.1186/1472-6750-7-78

**Published:** 2007-11-19

**Authors:** Jinny L Liu, George P Anderson, Ellen R Goldman

**Affiliations:** 1 Center for Bio/Molecular Science and Engineering, code 6920, Naval Research Laboratory, 4555 Overlook Ave. SW. Washington, DC 20375, USA

## Abstract

**Background:**

Shark heavy chain antibody, also called new antigen receptor (NAR), consists of one single Variable domain (V_H_), containing only two complementarity-determining regions (CDRs). The antigen binding affinity and specificity are mainly determined by these two CDRs. The good solubility, excellent thermal stability and complex sequence variation of small single domain antibodies (sdAbs) make them attractive alternatives to conventional antibodies. In this report, we construct and characterize a diversity enhanced semi-synthetic NAR V display library based on naturally occurring NAR V sequences.

**Results:**

A semi-synthetic shark sdAb display library with a complexity close to 1e9 was constructed. This was achieved by introducing size and sequence variations in CDR3 using randomized CDR3 primers of three different lengths. Binders against three toxins, staphylococcal enterotoxin B (SEB), ricin, and botulinum toxin A (BoNT/A) complex toxoid, were isolated from panning the display library. Soluble sdAbs from selected binders were purified and evaluated using direct binding and thermal stability assays on the Luminex 100. In addition, sandwich assays using sdAb as the reporter element were developed to demonstrate their utility for future sensor applications.

**Conclusion:**

We demonstrated the utility of a newly created hyper diversified shark NAR displayed library to serve as a source of thermal stable sdAbs against a variety of toxins.

## Background

Sharks, similar to camelids, possess unconventional heavy (H) chain antibodies, consisting of heavy chain homodimers in which each chain contains one single variable and five constant domains [1,2]. The conserved amino acid residues between the shark heavy chain antibody and those involved in forming the core of immunoglobulin and T-cell receptor variable regions, gave impetus for naming shark heavy chain antibody Immunoglobulin New Antigen Receptor (IgNAR or NAR) [2,3]. Structural analysis by electron microscopy, crystal structure, and 3D modeling revealed that there are three NAR isotypes. These isotypes are defined according to their pattern of inter-loop disulfide linkages within the variable region and the timing of their appearance during the animal's development [3-6].

Using genetic engineering a single NAR variable (V) antigen-binding domain can be expressed as a separate soluble protein often referred to as a shark single domain antibody (sdAb). Shark sdAbs contain four conserved frame regions (FRs) and two complementarity-determining regions (CDRs), making them the smallest (~12 kD) Ig-based recognition units with full capacity for antigen binding affinity and specificity. Due to their small size, they may be able to access antigen epitopes not generally recognized by recombinant conventional antibodies [7]. Although both shark and camelid sdAbs share similar structural features and functional requirements [3], shark sdAb lack a conventional CDR2, and contain two hyper-variable regions (HVs), HV2 and HV4, which may contribute to antigen binding [3,8,9]. According to the crystallographic analysis of NAR V structure, the loop of HV2, located within the FR2-CDR2 region, is located across the middle of the molecules and may influence the conformation of the CDR3; moreover, the loop of HV4, located between HV2 and CDR3, is formed proximal to CDR1 and may influence the antigen binding interactions [3].

Similar to camelid sdAbs, shark sdAbs exhibit excellent solubility for protein production, superior to many recombinant conventional antibodies, and retain conformational stability when heated or refold correctly upon cooling, [10-13]. These intrinsic properties make sdAb exceptional alternatives for diagnostic applications. Using phage display technology and PCR amplification, the repertoire of the naturally occurring NAR V from either immunized or naïve (non-immunized) animals were established and used for panning against target antigens [10,11]. High affinity binders to a specific target were obtained from immunized libraries however required a waiting period for suitable immunization to be achieved and an animal care facility [11]. On the other hand, weak binders against a wide variety of target antigens were obtained from naïve libraries; they were selected rapidly and no immunization period was required [10,14]. If higher affinity binders are desired than those obtained from the naïve library, the sdAb can be enhanced using *in vitro *affinity maturation [12,15].

It is believed that the diversity of naturally occurring NAR V results from multiple rearrangements of the CDR genes and somatic hypermutations *in vivo *[2]. Consistent with this finding, the complexity of shark NAR V usually resides in CDR1 with sequence variation within residues 28–33 and an extended CDR3, which varies in length (5–23 residues) and in amino acid composition. Routes to introduce diversity and increase the complexity of naïve libraries include: variation of CDRs via DNA shuffling [16], PCR using randomized primers, and random mutagenesis by error prone PCR [17] or dNTP analogs [18]. Although DNA shuffling, which involves the recombination of several small DNA fragments within a whole variable region, has been successfully used to create a semi-synthetic llama library with giga diversity [19], it has not been used for constructing a more diverse shark display library due to the short NAR V DNA fragments, less than 400 bp in size. Instead, PCR using randomized CDR3 sequences has been used to construct more diverse synthetic libraries, resulting in binders with higher affinity and specificity to target antigens [10,12].

Our previously described naïve NAR V library, SP, from spiny dogfish shark (*Squalus acanthias*) was successfully panned for the isolation of binders to cholera toxin; however our ability to obtain specific binders towards other targets was disappointing [20]. In this study, we expanded the utility of the naive display library by introducing variations in CDR3 amino acid composition and length (13, 16, or 18 residues). In this manner, a semi-synthetic NAR V display library, SPSL1, with a complexity of ~1e9 was successfully constructed and used for panning against ricin, staphylococcal enterotoxin B (SEB) and BoNT/A complex toxoid. We successfully selected binders against these three toxins and characterized the purified sdAbs using ELISA and Luminex 100 assays. Our results suggest that this new diversified NAR V display library can serve as a fruitful source of sdAbs against a variety of toxins.

## Results and Discussion

### Library construction and selections

A semi-synthetic shark display library was constructed starting with DNA isolated from a naturally occurring spiny dogfish shark library (SP) by randomizing CDR3. Most clones in the SP library had loop lengths between 13 to 18 amino acids based on 136 sequences (unpublished data). Three random primers (Table 1) were used in PCR reactions with the aim of increasing diversity of the new shark library by varying the length and residues within CDR3. These primers are designed to generate CDR3s with loop lengths of 13, 16, or 18 amino acids to target the dominant populations. Loop lengths of 14, 15, or 17 amino acids can be used in the future to introduce more diversity if necessary. Sequences from 13 random clones confirmed that CDR3 was composed of 13, 16, or 18 residues with random combinations of amino acids. Among these clones, 7 out of 13 clones appeared to have full-length sequences, 2 out of 13 had either frame shifts or deletion products, and 4 out of 13 clones contained amber codons. Since each of the 13 clones had a unique sequence, we estimated that the newly constructed shark library (SPSL1) contains ~1e9 individual clones, a complexity of ~1e9 with ~8e8 coding for full-length sdAb proteins. Compared to error prone PCR and DNA shuffling, randomized nucleotides primers generate a higher percentage of clones containing amber stop codons within the CDR3 region. However, since phage were prepared from an *E. coli *amber suppressor strain, the full- length proteins were still displayed on the phage tails during panning enrichment. Therefore, the presence of amber codons did not decrease the effective complexity of our new library.

**Table 1:** Randomized oligonucleotide primers used for amplification of randomized spNAR V CDR3 sequences.

Direction	Number	Sequence (5'-3')
Forward	Primer mix^a^	#8406, #8407/8, #7554/6, #9686/7, forextra
Reverse	Primer mix^b^	#6974/7, 7553/5, #9688/9, revextra
Reverse	spCDR3 R18	GGTYARWRSGGTKCCAKYTCC(MNN)_18_AGYKTTGCARWWAKACGTGGC
Reverse	spCDR3 R16	GGTYARWRSGGTKCCAKYTCC(MNN)_16_AGYKTTGCARWWAKACGTGGC
Reverse	spCDR3 R13	GGTYARWRSGGTKCCAKYTCC(MNN)_13_AGYKTTGCARWWAKACGTGGC

a, b were described in detail as in Liu *et al*. [20].

**Figure 1:**
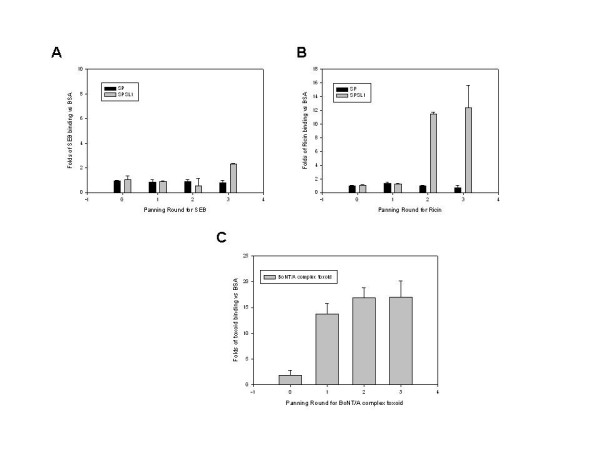
Polyclonal phage ELISA. Phage pools obtained from panning the new SPSL1 with the three tested toxins and panning the previously established SP with SEB and ricin were tested for binding specificity using ELISA. After three rounds of panning SPSL1, SEB binder phages were enriched approximately three-fold, while no enrichment was observed from panning SP (A). Ricin binder phages were enriched over ten-fold after second and third panning of SPSL1 and no enrichment was observed in panning SP (B). BoNT/A complex toxoid binders were enriched approximately twelve-fold after the first round and there was no significant enrichment after the second round. The same amount of the phage from each sample was used for the assay. Data were obtained from two independent experiments.

After three rounds of panning the SPSL1, we observed mild enrichment (~3-fold) for SEB binding (Fig. 1A) and strong enrichment (> 10-fold) for ricin. The binding enrichments from panning SPSL1 for both toxins were higher than those from panning the naturally occurring NAR V display library, SP (Figs 1A and 1B). In addition, we observed a 12-fold enrichment for BoNT/A complex toxoid binding after the first SPSL1 panning (Fig. 1C). For each toxin we screened 96 clones randomly selected from SPSL1 panning round 2 or 3 using monoclonal phage ELISA as described in the methods section. From each selection we sequenced 15 clones that had a ratio of toxin binding to BSA binding of greater than 3. We obtained two unique SEB binding sequences, one ricin binding sequence and two BoNT/A complex toxoid binding sequences.

### Sequence analysis

**Figure 2:**
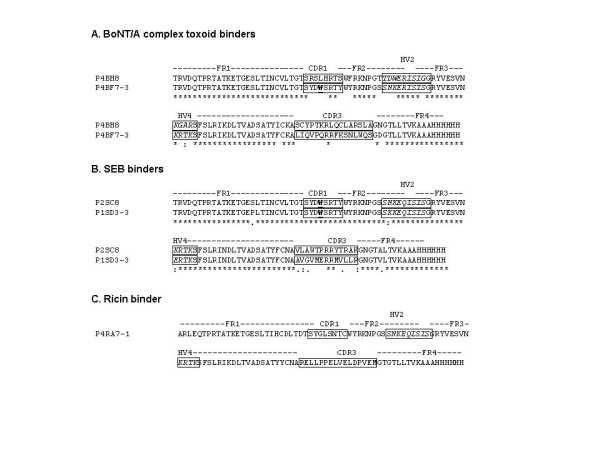
Sequences of isolated toxin binding sdAb. Fifteen potential binders for each tested target were sequenced. The resulting unique sequences were decoded into amino acid sequences, which were aligned using ClustalW and compared. Two BoNT/A complex toxoid binders had common R32, T33 in CDR1 (residue 28–35); E47, R48, I49, S50, I51 in HV2 (residue 44–53); K61 in HV4 (residues 61–65); and R92 in CDR3 (residues 86–101) (A). Two SEB binders shared identical CDR1, similar HVs except residues 4 and 61, and two conserved residues, R92 and R93 in CDR3 (residues 86–98) (B). The ricin binder had C33 in CDR1 and 16 residues in CDR3 (C). The homologous residues between binders were labeled with "*" and similar substitution with ":"and '.'. The CDRs and hyper variable regions are indicated within the box area. The invariant W31 within CDR1 in type 3 NAR V is in bold and underlined.

Ninety-six clones were screened for each selection and fifteen potential binders for each toxin were sent out for sequencing. Unique toxin binding sequence(s) were analyzed using ClustalW [21] (Fig. 2). The two BoNT/A complex toxoid binders, P4BH8 and P4BF7-3, varied significantly in amino acid composition within the four hypervariable regions, suggesting they target different epitopes. Interestingly, they both showed similar binding reactivity toward BoNT complex toxoid subtypes (Fig. 2A). The SEB binders, P2SC8 and P1SD3-3, shared identical CDR1, HV2, and conserved arginine within CDR3 but varied in amino acid 61 within the HV4 region (Fig. 2B).

The two SEB binders along with the BoNT/A complex toxoid binder, P4BF7-3, had no formation of inter-loop disulfide bonds and shared a conserved W31 within CDR1, which are major features of type 3 NAR V originally defined as appearing only in juvenile nurse sharks less than one year old [5,18]. Previously, we discovered that our spiny dogfish NAR V display library derived from adult animals over one year old contained approximately 7% of type 3 NAR V fragments. This may be a unique feature of spiny dogfish shark [20]. These type 3 spiny dogfish shark NAR V differ from ones found in nurse shark, since they exhibited more diverse length and composition with or without a conserved F96 in CDR3 [5,20,22]. Amino acid composition has a great influence on conformation and stability. As the spiny dogfish shark type3 NAR V binders lack the invariant aromatic Y86 and F96 found to participate in forming more rigid CDR3 loops, they likely exhibit different CDR conformational plasticity than type3 NAR V from nurse sharks [22]. Ricin binder, P4RA7-1 is an atypical type 2 NAR V with no formation of inter-loop disulfide bonds due to the lack of Cys in the CDRs (Fig. 2C). One of the BoNT/A complex toxoid binders, P4BH8, is an atypical type 1 NAR V that lacks Cys residues in FR2 and FR4 but contains two CDR3 Cys residues, which allows the formation of an intra-loop disulfide bridge to stabilize CDR conformation. The mechanism of CDR conformation stability for these atypical NAR V should be different from their corresponding typical NAR V and is a topic for future investigation. It is likely that HV2 and HV4 may play extremely dominant roles to stabilize CDR conformation [3,8,23].

### Expression of soluble monomeric SdAbs

Five selected binders were sub-cloned to the pEcan 22 expression plasmids, a gift from Dr. Andrew Hayhurst, for overproduction [24]. Monomeric soluble protein was purified using a nickel affinity column followed by gel filtration [20]. Expression levels for these five binders varied. Yields for P4BH8, P4BF7-3, and P4RA7-1 were about 1.0 mg per liter of bacterial culture; this is similar to the yield of hen egg white lysozyme binders isolated from a repertoire of nurse shark NAR V [11]. The production amounts for the two SEB binders, P1SD3-3 and P2SC8, were about 6-fold less. The difference in expression levels may be due to overall conformation stability and domain surface charges resulting from sequence and structural diversity. However, we did not observe a good correlation pattern between the clone sequences and the expression levels based on the comparison of amino acid residues. Structural and 3D modeling studies for these clones are required to reveal more information about correlations between protein expression level and sequence variation.

### Direct binding to determine affinity and specificity

Binding affinity and specificity was determined using direct binding assays with toxoid-coated and toxin-coated microspheres as described in the methods. The equilibrium constant (k*d*) for each sdAb and conventional antibody was calculated from direct binding curves using the formula, y = (Bmax)x/(Kd+x) (Table 2). This calculation let us compare the sdAb and conventional antibodies. Overall, the affinities of the selected binders were in the 100's of nM, many times that of conventional antibody, however they could be used as scaffolds for further affinity maturation studies (Table 2).

**Table 2:** Equilibrium dissociation constants

**Ricin**	**K*d *(nM)**
*sdAb-P4RA7-1*	299 ± 49
*Cy3-Rab-Anti-Ricin IgG*	50.0 ± 7.5
**SEB**	
*sdAb-P1SD3*	10.2 ± 7.2
*sdAb-P2SC8*	107 ± 38
*Cy3-sh-anti-SEB IgG*	3.6 ± 3.6
**BoNT/A complex toxoid**	
*sdAb-P4BF7-3*	154 ± 26
*sdAB-p4BH8*	390 ± 90
*Rab-anti-BoNT*	0.02 ± 0.003
**BoNT/B complex toxoid**	
*sdAb-P4BF7-3*	106 ± 22
*sdAb-P4BH8*	332 ± 68
*Rab anti-BoNT*	0.05 ± 0.007
**BoNT/E complex toxoid**	
*sdAb-P4BF7-3*	93 ± 18
*sdAb-P4BH8*	309 ± 75

**Figure 3:**
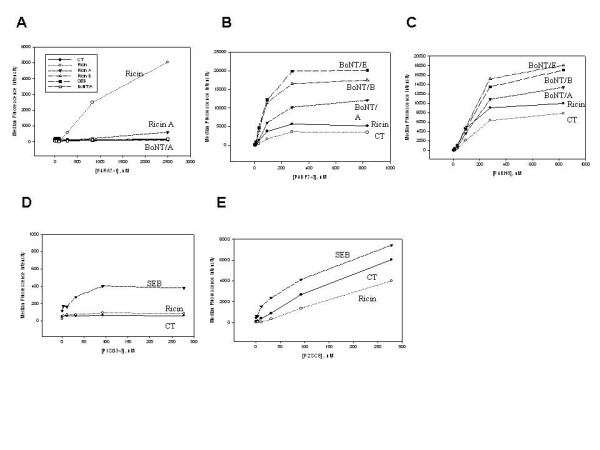
Luminex direct binding assay. Microspheres with covalently attached toxins and irrelevant targets were mixed with purified sdAbs at concentrations of 0, 3.4, 10.9, 30.9, 92.6, 277.8, 833.3, and 2500 nM to test the binding affinity. Microspheres were sorted using the Luminex 100 and the median fluorescence signals were obtained. The ricin binder showed that a maximal binding concentration (Vmax) for ricin and ricin A was around 300 nM with < 0.1% of non-specific binding to ricin B, CT, SEB and BoNT/A complex toxoid (A). BoNT/A complex toxoid binders, P4BF7-3 and P4BH8, exhibited the same Vmax. P4BF7-3 had approximately 13% and 25% of the non-specific binding signal to CT and ricin, respectively (B) and P4BH8 had even higher non-specific binding signals than P4BF7-3 (C). SEB binder P1SD3-3 had Vmax around 10.9 nM with less than 1% of non specific binding signals to CT and ricin (D), while P2SC8 had Vmax around 300 nM with more than 30% of non-specific binding signals (E).

The isolated ricin binder, P4RA7-1, bound specifically to intact ricin and ricin A relative to irrelevant targets, CT, BoNT/A complex toxoid and SEB, suggesting it is a ricin A chain binder (Fig. 3A). Its superior specificity makes it an ideal candidate for further affinity maturation. One of the BoNT/A complex toxoid binders (P4BF7-3) was fairly specific to BoNT complex toxoid subtypes, but the other (P4BH8) showed significant non-specific binding to ricin and CT (Figs. 3B and 3C). The measurement of direct binding for the ricin binder (Fig. 3A) and CT binders isolated previously [20] (data not shown) to microspheres with BoNT complex toxoids immobilized to their surface showed no significant interaction, indicating that the sdAb do not as a class nonspecifically bind BoNT complex toxoids. Thus, the non-specific binding observed for any binder selected appears to be intrinsic to just that sdAb, however it points out the necessity to critically evaluate specificity for binders derived by this or similar methods. Nonetheless, for the BoNT binders selected, both had stronger binding to the three tested BoNT complex toxoid subtypes, E, B, and A than to irrelevant targets (Fig. 3B). Generally, they appeared to bind subtypes E and B even more than A (Figs. 3B and 3C), suggesting that toxoid complexes derived by formalin-inactivation may share some common epitopes among subtypes (Figs 3B and 3C). Since P4BF7-3 specifically cross-reacts to other complex toxoid subtypes it may be useful for generic BoNT detection, as opposed to subtype classification. The anti-BoNT sdAb were also tested for their binding to a range of BoNT subtypes using the various toxins as wells as their toxoids immobilized on microspheres. Both BoNT/A binders showed considerable cross-reactivity to other BoNT subtypes; this binding was significantly greater than binding to irrelevant targets (preliminary data not shown). To obtain specific BoNT/A toxin binders, more library screening may be necessary; however these selected binders may find use for sample enrichment purposes, a critical need for such a potent toxin.

The SEB binders showed very different binding affinity and specificity despite their identical CDR1 and homologous CDR3 sequences (Figs. 2B, 3D and 3E). P1SD3-3 bound to SEB specifically relative to irrelevant targets, while P2SC8 exhibited more non-specific sticky interactions with irrelevant targets, CT and ricin (Figs 3D and 3E). Moreover, P1SD3-3, has ten-fold better binding affinity (k*d *= 10.2 nM) than P2SC8 (k*d *= 106.8 nM) (Table 2). A recent affinity maturation study showed that changing S61 to R61 within the HV4 in nurse shark NAR V contributed to increased binding affinity resulting from more van der Waals' contacts on the mature sdAb [8]. The change of K61 to E61 within HV4 in P1SD3-3 might exhibit a similar effect and perhaps resulted in observed higher binding affinity and specificity of P1SD3-3 compared to P2SC8 (Fig. 2B, Figs. 3D and 3E). P2SC8 and P4BF7-3 had identical sequences for most regions except CDR3 and amino acid 48 in HV2. The resulting differential binding reactivity to all tested toxins suggests that CDR3 alone may play a major role to determine the binding specificity in these two clones.

### Thermal stability determinations

**Figure 4:**
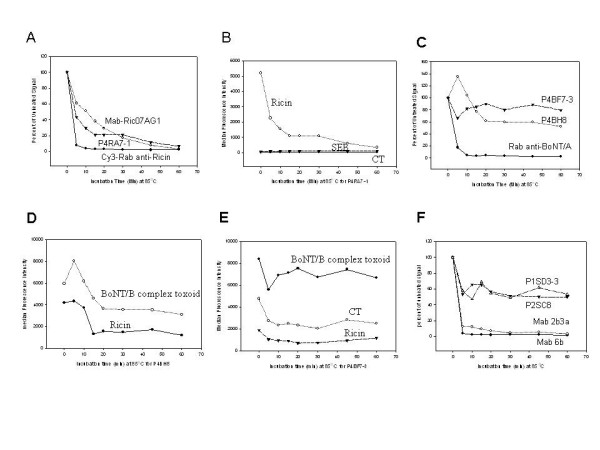
Thermal stability assay. Antibodies were heated at 85°C for various time points up to 60 min and cooled down prior to measuring binding affinity and specificity using luminex direct binding assays. Each antibody at a concentration of maximal binding mentioned in Fig. 3 was used for this thermal stability assay. Mab-Ric07AG1, and ricin binder, P4RA7-1, retained 60% and 40% of the original activity, respectively; while Cy3-Rab anti-Ricin had only 10% of remaining activity upon 5 min of heating (A). During the heating, P4RA7-1 binding to irrelevant targets, CT and SEB, was insignificant. (B). After 5 min of the heating, P4BH8 and P4BF7-1 had 130% and 60% of the untreated activity, while Rab anti-BoNT/A retained 20% of the untreated activity. Upon 60 min of heating, both retained 60%–80% of untreated activity, while Rab anti-BoNT/A lost almost 100% of the initial activity within the first 10 min of heating. (C). P4BH8 decreased 75% of the initial non-specific binding to ricin after 15 min of heating (D). P4BF7-1 decreased 50–60% of the initial non-specific binding to irrelevant target(s), ricin and CT, after 10 min of heating (E). Both P1SD3-3 and P2SC8 retained about 55% of the initial activity after 5 min of heating, while two conventional antibodies, Mab 2b3a, and Mab 6b, lost about 90–95% of the initial activity (F).

Our results indicated that the ricin binder showed better thermal stability than polyclonal antibody, and was similar in stability to the monoclonal antibody tested (Fig. 4A). Upon heating, the ricin binder still retained its specificity showing insignificantly binding to irrelevant targets, CT and SEB (Fig. 4B). Both BoNT/A complex toxoid binders retained a substantial amount (60%–80%) of the original binding activity after being heated for 60 min at 85°C, while non-specific binding was actually reduced over the same period (Figs 4D and 4E). Overall, they showed much better thermal stability than conventional antibody, which lost activity rapidly upon heating (Figs, 4C, 4D and 4E).

Of the two, P4BF7-3 appeared to be more stable than P4BH8 (Fig. 4C), suggesting that it might have a more stable CDR conformation. The conformational stability may partially result from the formation of an intra-loop disulfide bridge within CDR3 (Fig. 2A). Interestingly, P4BH8, showed 30% more than initial binding reactivity to target, upon heating for 5 min (Fig. 4C). This phenomenon is often seen in sdAbs, possibly due to better binding of the refolded structure that results from a short period of heating and rapid cooling; this same phenomenon may also be the cause for improvements in specificity [12,13,20].

Both SEB binders exhibited better thermal stability than tested conventional antibodies (Fig. 4F). They showed similar thermal stability profiles, suggesting both share a similar CDR conformation resulting from similar sequences (Fig. 2B).

### Use of sdAb as reporter reagents in sandwich assays

**Figure 5:**
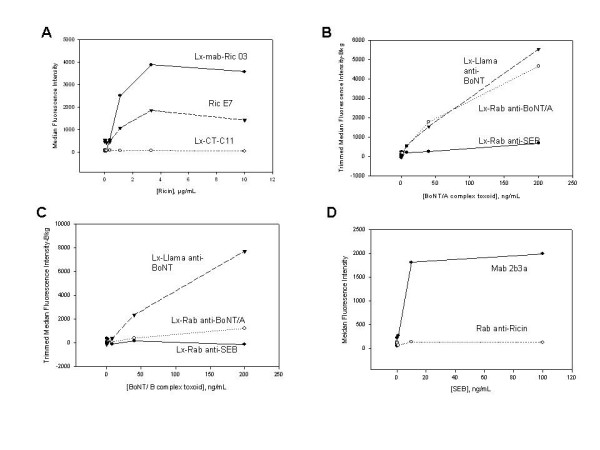
Luminex Sandwich assay. To measure the utility of sdAbs as tracer antibody, luminex sandwich assays were conducted. Purified antibodies for each tested target along with Ni-SA-PE were mixed with toxins captured by microspheres coated with the capture antibodies. Mab-Ric 03, llama sdAb Ric E7, and llama sdAb CT-C11 (as negative control) were used to capture ricin, which was at concentrations of 0.1–10 μg/mL. Ricin binder, P4RA7-1, along with Ni-SA-PE were used as a tracer and were able to detect ricin at 1 μg/mL captured by Mab-Ric 03 and llama sdAb Ric E7, but not negative control (A). Llama anti-BoNT, Lx-Rab-anti-BoNT/A and Lx-Rab anti-SEB (negative control) were used to capture BoNT/A complex toxoid, which was at concentrations between 0.1 to 200 ng/mL. P4BF7-3 and Ni-SA-PE were used as tracers to detect BoNT/A complex at a minimum concentration of 40 ng/mL captured by Llama anti-BoNT, Lx-Rab-anti-BoNT/A, but not Lx-Rab anti-SEB (B). The same tracers were used to detect BoNT/B complex toxoid at a concentration of 40 ng/mL captured by only Llama anti-BoNT, but not by Lx-Rab-anti-BoNT/A or Lx-Rab anti-SEB (C). Mouse monoclonal antibody against SEB, Mab2b3a, and Rab anti-Ricin (negative control) were used to capture SEB, at concentrations range from 0.01 to 100 ng/mL. P1SD3-3 and Ni-SA-PE were used as tracer to detect the SEB at a minimum concentration of 10 ng/mL, captured by Mab2b3a, but not by Rab anti-Ricin (D).

Sandwich assays using sdAb in conjunction with Ni-SA-PE as tracer reagents were performed as detailed in the methods. Capture antibodies including irrelevant antibody as a negative control, conventional antibodies and sdAbs were used. Ricin binder, P4RA7-1, was able to detect ricin at a concentration of 1 μg/mL, using monoclonal antibody or llama sdAb Ric E7 as the capture reagents and showed no binding to the negative control beads coated with CT antibody, Lx-CT-C11 (Fig. 5A). For BoNT sandwich assays, llama anti-BoNT was used as the capture reagent for either BoNT/A or B complex toxoids, which was subsequently detected at a concentration of 40 ng/mL by P4BF7-3 using Ni-SA-PE to generate signal (Figs. 5B and 5C). As might be expected, the Rab anti BoNT/A showed good selectivity, capturing BoNT/A complex toxoid, but not B complex toxoid (Fig. 5B and 5C). For SEB sandwich assays, the specific SEB binder, P1SD3-3 was used as a tracer and could detect SEB at a concentration of 10 ng/mL with insignificant non-specific signal observed on the control microspheres (Fig. 5D). In general, we were able to demonstrate the use of sdAbs as reporter reagent in sandwich assays.

## Conclusion

The sdAbs selected from the new randomized naïve shark library exhibit serviceable specificity and excellent thermal stability, but their binding affinity was weaker than the conventional antibodies tested. Often, affinity maturation is required to obtain high affinity binders. Besides the common approaches described in the background section, 3D structural modeling also provides a powerful tool to reveal more detailed information regarding the change of amino acids within the constant regions and HVs [9]. However, the typical increase in binding affinity has been only 10-fold using these approaches, which would still not produce binders comparable to the best conventional antibodies [9]. Alternatively, multiple copies of sdAbs against the same target can be linked together to generate high avidity structures [2,25,26], which can result in three orders of magnitude higher affinity than the monomeric sdAb. This may be a more attractive way to improve binding reactivity for our sdAb (Table 2).

In the future, we may need to introduce more variation in the CDR3 loop length in combination with the preferred insertion of Cys into defined CDR3 positions. Construction of a more diverse library, containing a greater percentage of conventional type 1 and 2 NAR sequences may allow us to obtain specific binders for each BoNT subtype for detection purpose. Nevertheless, our results indicated that the new semi-synthetic shark library had better CDR3 diversity and better utility than our previously established naturally occurring NAR V display library, suggesting this may be the correct path towards obtaining a limitless source of sdAbs against a variety of toxins for sensor applications.

## Methods

### Library construction

DNA template was isolated from the naturally occurring shark library using the plasmidpure DNA miniprep kit (Sigma, St. Louis, MO) and was used to amplify randomized CDR3 fragments with forward primer mix and randomized reverse primers (table 1). PCR condition was as follows: 94°C for 30s, 55°C for 90s, and 70°C for 30s, and 35 amplification cycles. The resulting 400 bp PCR products were then used as templates to amplify the full length NAR V fragments flanked with *Sfi*I and *Not*I restriction sites on 5' and 3' respectively using forward and reverse primer mixes [17]. The full length NAR V fragments and pHen2 plasmids were then cut with *Sfi*I and *Not*I, followed by overnight ligation at 15°C. The ligation mixture was cleaned by Qiagen PCR kit (Qiagen, Valencia, CA) and subjected to electroporation using XL 1 Blue cells (Strategene, La Jolla, CA). The cells were plated on big Nunc Bio-assay dishes containing LB agar, 2% glucose, and 100 μg/mL ampicillin and grown overnight. The transformed bacteria were scrapped and stored at -80°C the next day. The resulting semi-synthetic library was named SPSL1.

### Toxin binder selection by panning and ELISA

Panning and ELISA assays were carried out essentially as described previously [20]. Ricin, SEB and BoNT/A complex toxoid at concentrations of 10 μg/mL were passively immobilized on high binding 96 well plates and used to mine the semi-synthetic shark NAR V display library, SPSL1, in 3 rounds of panning. Polyclonal phage ELISAs on target and control antigens were used to identify rounds in which clones had been successfully enriched. Monoclonal ELISAs were subsequently used to identify monoclonal sdAbs [20]. Typically, 15 antigen positive clones were subjected to DNA sequencing to identify unique clones.

### Expressing and purifying sdAb

NAR V fragments isolated from potential binders were cut with *Sfi*I and *Not*I. The resulting fragments were inserted into pEcan 22 vectors and transformed into the *E. coli *Tuner strain for protein expression. Expressed protein was purified by osmotic shock from Tuner cultures, immobilized metal affinity chromatography, and gel filtration [20,27].

### Characterizing purified sdAb by Luminex 100 immunoassays

For direct binding assay, Luminex microspheres coated with relevant and irrelevant toxins were incubated with purified sdAbs at the concentrations of 0.01–2500 nM for 30 min, followed by the addition of fluorescent tracer, Ni-streptavidin-phycoerythrin (Ni-SA-PE). For thermal stability testing, purified sdAb proteins and control antibodies were heated to 85°C for 60 min with samples removed to cooling at various time points and analyzed for antigen binding activity. In all analyses, sdAb binding to irrelevant antigens was also monitored to ensure signal was not due to non-specific binding of unfolded sticky protein. For sandwich assay, Luminex microspheres with antibodies immobilized were mixed with target antigens. Then a second sdAb, and Ni-SA-PE were added into the reaction to measure the binding of captured antigens. The preparation of immuno-reagents was described previously [19].

## Authors' contributions

JL designed the studies and the strategy to introduce the complexity into CDR3 and developed the semi-synthetic hyper-diversified shark SPSL1 library. She selected binders against toxins, purified sdAbs and drafted the manuscript. GA performed the binding assays and helped to draft the manuscript. ERG designed the studies, helped to draft the manuscript and guided the overall project. All authors read and approved the final manuscript.
